# *pypet*: a python toolkit for simulations and numerical experiments

**DOI:** 10.1186/1471-2202-16-S1-P184

**Published:** 2015-12-18

**Authors:** Robert Meyer, Klaus Obermayer

**Affiliations:** 1 Neuroinformatics Group, Technische Universitaet Berlin, 10587 Berlin, Germany; 2 Bernstein Center for Computational Neuroscience Berlin, 10115 Berlin, Germany

## 

"*pypet*" (python parameter exploration toolkit [1]) is a new multi-platform python toolkit for management of simulations and storage of numerical data. Exploring or sampling the space of model parameters is one key aspect of simulations and numerical experiments. *pypet *was especially designed to allow easy and arbitrary sampling of trajectories through a parameter space beyond simple grid searches. Moreover, special focus is put on managing different neuron models in python network simulations like BRIAN [2]. Simulation parameters as well as the obtained results are collected by *pypet *and stored in the widely used HDF5 file format [3]. This allows fast and convenient loading of data for further analyses.

Furthermore, *pypet *provides an environment with various features. For example, among these are multiprocessing for fast parallel simulations, dynamic loading of data, integration of Git version control, and supervision of experiments via the electronic lab notebook Sumatra [4]. A rich set of data formats is supported, encompassing native python types, numpy and scipy data, pandas DataFrames [5], and data from BRIAN [2]. Moreover, the toolkit is easily extendible to allow the user to add customized data formats. *pypet *is a very flexible tool and suited for short python scripts as well as large scale projects. Thus, *pypet *supports reproducible research in computational neuroscience and other disciplines that involve simulations and numerical experiments.
